# Additive manufactured push‐fit implant fixation with screw‐strength pull out

**DOI:** 10.1002/jor.23771

**Published:** 2017-11-22

**Authors:** Richard J. van Arkel, Shaaz Ghouse, Piers E. Milner, Jonathan R. T. Jeffers

**Affiliations:** ^1^ Department of Mechanical Engineering Imperial College London London SW7 2AZ United Kingdom

**Keywords:** initial implant stability, press‐fit, minimally invasive implants, porous implants, 3D printing

## Abstract

Additive manufacturing offers exciting new possibilities for improving long‐term metallic implant fixation in bone through enabling open porous structures for bony ingrowth. The aim of this research was to investigate how the technology could also improve initial fixation, a precursor to successful long‐term fixation. A new barbed fixation mechanism, relying on flexible struts was proposed and manufactured as a push‐fit peg. The technology was optimized using a synthetic bone model and compared with conventional press‐fit peg controls tested over a range of interference fits. Optimum designs, achieving maximum pull‐out force, were subsequently tested in a cadaveric femoral condyle model. The barbed fixation surface provided more than double the pull‐out force for less than a third of the insertion force compared to the best performing conventional press‐fit peg (*p *< 0.001). Indeed, it provided screw‐strength pull out from a push‐fit device (1,124 ± 146 N). This step change in implant fixation potential offers new capabilities for low profile, minimally invasive implant design, while providing new options to simplify surgery, allowing for one‐piece push‐fit components with high levels of initial stability. © 2017 The Authors. *Journal of Orthopaedic Research®* Published by Wiley Periodicals, Inc. on behalf of the Orthopaedic Research Society. J Orthop Res 36:1508–1518, 2018.

Metal implants are used to treat orthopaedic trauma and disease around the body and include spinal fixators, bone fracture fixation plates, tendon repair anchors, ligament reconstruction fixation screws, chondral repair implants, and total joint replacement implants. These implants are used in high volumes—for example >100,000 anterior cruciate ligament (ACL) reconstructions are performed in the US each year,^1^ while in the UK >200,000 joint replacement procedures are performed annually,^2^ with numbers projected to rise. It is estimated that 7 million Americans are currently living with a joint replacement.^3^

Implant fixation is critical to success of these procedures; indeed loosening is a primary reason for failure of joint replacement implants.^2^, ^4^ Modern implants typically rely on press‐fit or screw fixation. Screws are often used for ACL surgery, dental implants, fracture fixation, and early intervention chondral repair implants. They offer high levels of implant stability, with high pull‐out loads (around 1,000 N),^5^, ^6^ and by changing the thread, can be optimized for different bone densities.^7^ However, because screws must be rotated about their own axis to achieve fixation, they are of no use for non‐axisymmetric/multiple fixation features. Such designs either require modularity^8^ or press‐fit fixation.^9^, ^10^ Modularity between screw fixation bases and other components^8^ can lead to problems with intraoperative assembly and fretting/corrosion wear; the subsequent soft tissue reactions to the resulting metal debris can cause a severe revision burden.^11^ Press‐fit fixations enable non‐axisymmetric/multiple fixation features and are frequently used for arthroplasty components.^12^, ^13^, ^14^, ^15^ However, they provide lower initial fixation strength than screws (only around 50–150 N of pull‐out force, an order of magnitude less than a screw equivalent).^5^, ^6^, ^9^, ^16^, ^17^ A technology that is able to provide screw‐strength fixation, while allowing non‐axisymmetric/multiple fixation features could therefore enable new/improved orthopaedic treatments.

Improvements in implant fixation could come from additive manufacturing (AM) technology which has influenced a number of fields ranging from aerospace to sports equipment to orthopaedics as it offers engineers new design freedoms.^18^, ^19^ AM technology has been used for dental implants,^20^ mass market joint replacement designs,^12^, ^13^ and custom implants for the treatment of osteosarcoma.^21^ For implant fixation, a big draw of AM technology is the ability to create porous structures that bone can grow into, allowing improvement in long‐term fixation; consequently porous structures have been extensively researched.^22^, ^23^, ^24^, ^25^, ^26^, ^27^, ^28^, ^29^, ^30^, ^31^, ^32^ Recent research has also suggested that AM fixation features could improve the initial stability of implants.^14^, ^15^ This area has been much less researched but is of equal importance as initial implant stability is a prerequisite for long‐term fixation.

Given that AM has already been adopted as a viable manufacturing method for metal implants, there may be an opportunity to take advantage of its ability to create fixation surfaces that are not possible by conventional subtractive machining or forming. As metal implants are widely used around the body, this could benefit a large number of patients. Therefore, the aim of this research was to exploit the design freedoms of additive manufacturing to develop a linearly inserting fixation surface that anchors in bone, thereby attaining screw‐level pull‐out strength from a push‐fit device.

## METHODS

### Study Design

This study used a push‐in/pull‐out model to assess initial implant fixation. To allow comparison between greatly varying designs the implants were simplified to Ø8 × 16 mm cylinders. This was based on pegs currently used in total knee replacements,^9^ common bone screw sizes,^5^, ^6^ and established research examining press‐fit fixation.^17^, ^33^ Samples were cannulated reflecting common guide‐wire surgical implantation techniques and allowing for ease of mounting to a materials testing machine.

Three variations of pegs were explored: (i) solid Ti pegs with a rough surface, representing solid Ti implants with an applied porous coating^9^, ^34^, ^35^; (ii) porous structure pegs, representing fully porous implants^26^, ^36^; and (iii) our new barbed fixation pegs with dedicated, additive manufactured fixation features. ACL interference screws (Smith and Nephew, UK) of an equivalent size were also tested as a control.

The large design space was first narrowed through testing in synthetic bone foam (*N* = 4 per sample), a readily available porous medium that has mechanical properties equivalent to trabecular bone and low inter‐specimen variation. The version of designs A, B, and C with the largest pull‐out force were then further tested in a cadaveric tissue model (*N* = 8 per sample). A testing overview chart has been included in the Supplementary material (Fig. S1).

### Specimen Design, Manufacture, and Inspection

#### Design A: Solid Peg Fixation

Surface roughness was created by adding randomized sinusoidal‐based outer contours to the part with a target peak‐to‐trough roughness, *R*_z_, >500 μm, representing a typical bone fixation surface applied to traditional arthroplasty designs. The sinusoidal aspect allowed control of average amplitude and peak‐to‐peak spacing, while the randomization added local variation to the surface. The pegs were then manufactured in two shapes, first as the standard test shape straight sided cylinder (Ø8 × 16 mm) then as a tapering cylinder (1.72° half angle, minimum Ø8 mm).

Roughness was then measured with white light interferometry (Wyko NT9100, Veeco, UK). Samples were measured at 5× effective magnification, stitching 28 measurements across a 2 × 10 mm rectangle along the length of the peg. Roughness values were corrected for the geometry of a sample.

#### Design B: Porous Peg Fixation

Porous pegs were designed to be a stochastic (randomized) porous lattice structure.^22^ The effective elastic modulus of a peg could influence its fixation mechanics as it determines the ratio of implant/bone deformation following push in. Therefore, the pegs were manufactured with two target moduli spanning the range of trabecular bone: A low effective modulus structure of 600 MPa representing lower end of cancellous bone,^37^ and a high modulus structure of 2.6 GPa representing the modulus achieved by modern porous arthroplasty designs^25^, ^38^, ^39^ and the higher end of subchondral cancellous bone.^37^ The higher modulus was achieved through thicker struts rather than a change in the stochastic structure to isolate the effects of varying modulus. These two porous pegs were manufactured straight sided and tapered (1.72° half angle, minimum Ø8 mm) generating four designs.

Porosity of the stochastic structures was measured using a balance (accuracy ± 0.001 g, EL‐200S, Setra Systems, MA) and dividing by the mass of a solid equivalent (density of 4.42 g/cm^3^). Mechanical properties (modulus/compressive strength) were quantified by manufacturing *N* = 6 porous pegs without their mounting core and performing quasi‐static compression testing in an axial direction according to BS ISO 13314:2011 with the following two deviations: First, the pegs were directly measured rather than creating an equivalent structure sized according to the ISO standard, and second, compressive strength rather than plateau stress was used to define the σ_20_–σ_70_ hysteresis loop due to the brittle failure mechanism of titanium porous structures.^22^

#### Design C: Barbed Fixation Surfaces

**Figure 1:**
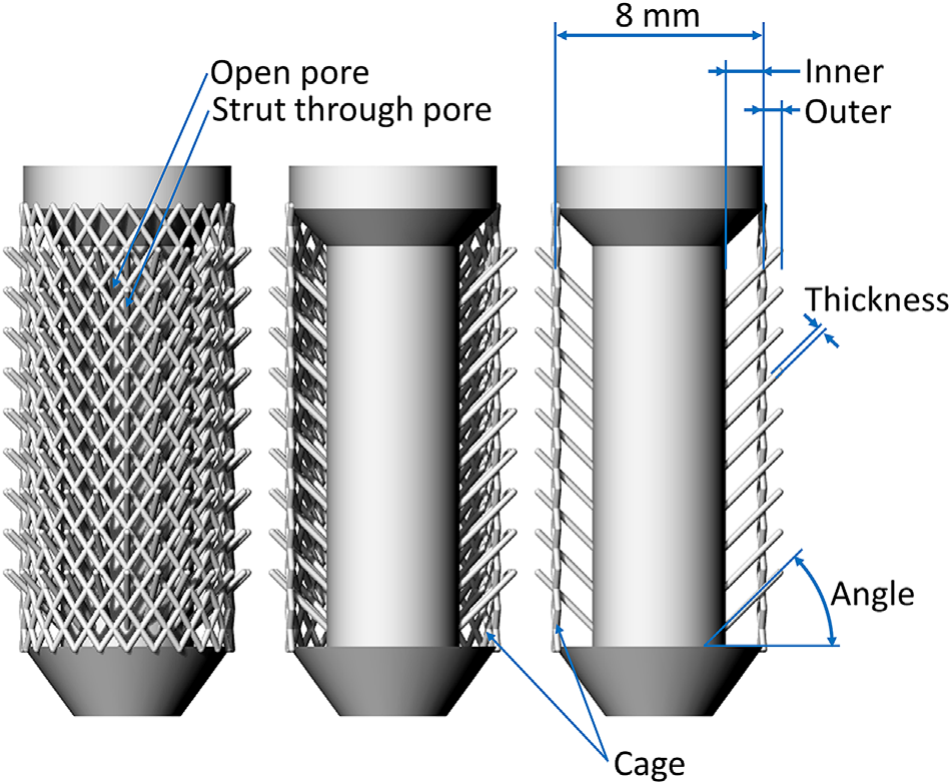
Diagram of the barbed fixation implant. From left to right the cage and front and rear fixation struts have been progressively removed to expose the design. Properties that effect the strut flexibility (inner and outer length, width, and angle) are highlighted, as are design features such as projecting the strut through a pore.

The design rationale for the barbed fixation surfaces was to make small, hook‐like directionally biased features on the surface of an implant. Being directionally biased, these struts could flex into the implant upon insertion, decreasing the interference with bone, providing low push‐in resistance, while they could then flex outwards and grip onto the bone under pull out, increasing interference and providing a high stabilizing force. Additive manufacture allows each strut to have increased length into the body of the implant (Fig. 1). This increases the available deflection of the strut upon insertion, while maintaining the same interference fit.

This fixation mechanism relies on the flexibility of the strut and therefore all variables that could affect the strut flexibility were examined. These included: Outer and inner length, thickness and angle. The number of fixation struts was also considered. Figure 1 shows the baseline specimen used and Table 1 details the variations made to this specimen. For each design variant, the optimum (maximum pull‐out force) was noted, and then combined to make a best‐case design which was used for comparison against other pegs and in the cadaveric knee tests.

**Table 1:** Design Variants for the Barbed Fixation Design Shown in Figure 1

Property	Baseline Specimen	Range
Inner radial length (mm)	1.5	0–2
Outer radial length (mm)	0.5	0–2
Angle (°)	45	15–75
Strut thickness (mm)	0.23	0.15–0.50
Strut density on core (#/mm^2^)	0.9	0–9

Flattened specimens (maintaining key dimensions) were also produced and a video method was used to confirm that the struts do indeed flex during implant insertion as intended.

#### CAD Workflow and Manufacture

All specimens were designed in Rhinoceros (v5, Robert McNeel & Associates Europe, Spain). Depending on the geometry, parts were sliced with one of two programs: Bulk solids (e.g., solid/tapered cylinders) were generated as 3D objects and sliced from STL files using Magics (v19, Materialise, UK). However, complex geometries result in large STL file sizes. Therefore, roughness contours, barbed fixation struts, and porous pegs were defined as lines, and were sliced with Material Engine (v1, Betatype Ltd, UK). These lines only gain thickness during the laser melting process, as previously described,^22^ thereby avoiding the need to generate complex STL models and thus saving on computation expense. Slices were uploaded to a metal powder bed fusion additive manufacturing system (AM250, Renishaw PLC, UK) which manufactured parts in 50 μm layers from Titanium spherical powder (Ti6Al4V ELI, particle size range 10–45 μm, D50: ∼27 μm, LPW Technology, UK).

### Testing

#### Synthetic Bone Tests

Synthetic cancellous bone blocks (20 PCF rigid cellular foam, size 130 × 180 × 40 mm, Model #1522‐12, Sawbones, Sweden) with a density of 0.32 g/cm^3^ were pillar drilled with holes spaced 25.4 mm (centre–centre) apart resulting in a 6 × 4 array of holes in each block. This spacing exceeds the two‐diameter minimum spacing recommended in previous research to mitigate any effects of foam block damage from adjacent tests.^40^ Solid and porous pegs (Designs A and B) were tested in holes with increasing interference fit: In 0.2 mm radial increments until a peak pull‐out load was obtained. Barbed fixation surface specimens (Design C) were tested in Ø8 mm holes with changes in interference being governed by the outer length of the fixation struts.

**Figure 2:**
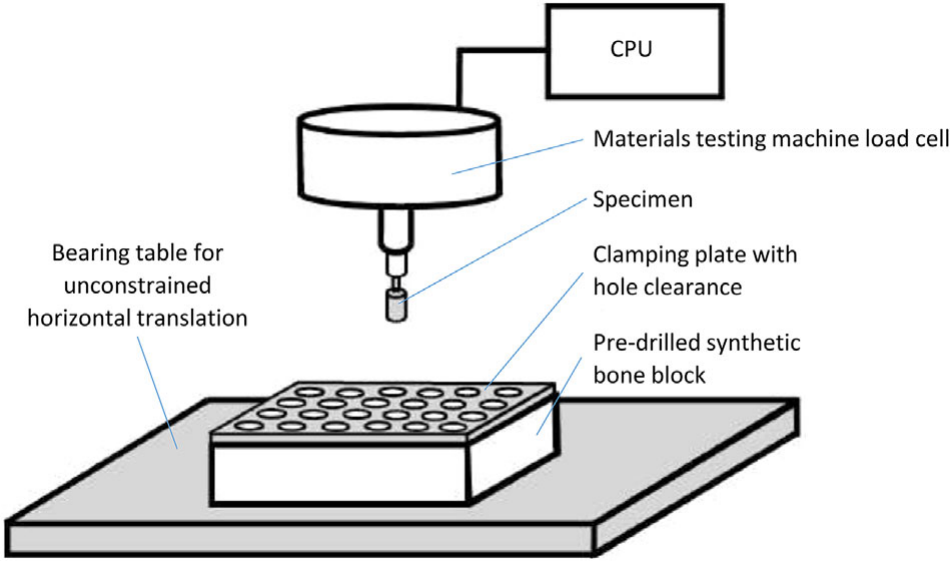
A schematic drawing of the synthetic bone tests highlighting the main features of the test set‐up.

The sawbones block was clamped with a metallic plate, sized to match the sawbones blocks, to the material testing machine (Fig. 2). This plate (with co‐located 25.4 mm spaced Ø16 mm holes) spread the clamping load over the entire surface of the sawbones testing block while providing sufficient hole clearance (always >2 mm) to prevent clamping stress from interfering with the test.^41^ A low friction bearing table allowed for unrestricted translation in the horizontal plane during all tests.

Specimens were then attached to the material testing machine actuator head and were pushed in vertically at a rate of 1 mm/s. There was a 10 s pause before specimens were then pulled out at 1 mm/s. This loading rate was within the range of previous research which found that pull‐out force was not affected by displacement rates between 0.01 and 10 mm/s.^17^ For each peg design/interference fit combination, *N* = 4 samples were tested, with each repeat assigned a fresh hole in a different synthetic bone block.

**Figure 3:**
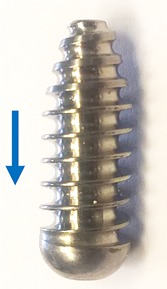
The ACL interference screw tested. The arrow indicates the direction of push out.

The set‐up was also used to test the push‐out force for a Ø9 × 20 mm titanium alloy (Ti6Al4V) ACL interference screw. This screw had a tapered thread for ease of insertion meaning that the working thread length of the screw provided an appropriate control for the cylindrical pegs. *N* = 4 screws were inserted manually into two hole sizes: Ø8 and Ø6.4 mm. The former was the recommended size for the screw which by design would also accommodate an ACL graft (which was not present in this test). Therefore the latter, equal to the minor diameter of the thread, was also tested. The screws’ cannula diameter was too small for our test fixture mounting and therefore screws were pushed out by the materials testing machine rather than pulled out. All other testing conditions (hole clearance >2 mm, hole spacing >25.4 mm, and rate 1 mm/s) were maintained. The direction of push out (Fig. 3) represented the typical direction of tension for a femoral ACL screw.

#### Knee Condyle Tests

Ethical approval for the tests was obtained from the host institution's tissue bank. Four fresh‐frozen cadaveric distal femora (two male, mean ± S.D. age 49 ± 9, range 37–58) were tested. Pegs were tested in both the medial and lateral condyles resulting in a sample size of *N* = 8 for each peg design.

Each femur was defrosted, dissected free of soft tissue and potted in a metal cylinder in a neutral position, defined as when the femoral shaft aligned vertically in the sagittal plane and when a spirit level across the condyles indicated they were flat in the coronal plane. Two holes were drilled into both the medial and lateral condyles (sized according to the optimums established in the sawbones tests) spaced 20 mm centre–centre with a minimum distance of 10 mm to the cortex. This replicated a set‐up previous used^9^ however in the present study, an arthroplasty bone cut was not made so that specimens were more representative of early intervention surgery for cartilage defect repair/ligament reconstruction than arthroplasty. Testing order/position bias was overcome by pairing holes such that the anterior medial and the posterior lateral were prepared for one peg type, and the posterior medial and anterior lateral hole for the other, then between specimens each peg type was tested in each specimen and in each position an equal number of times.

**Figure 4:**
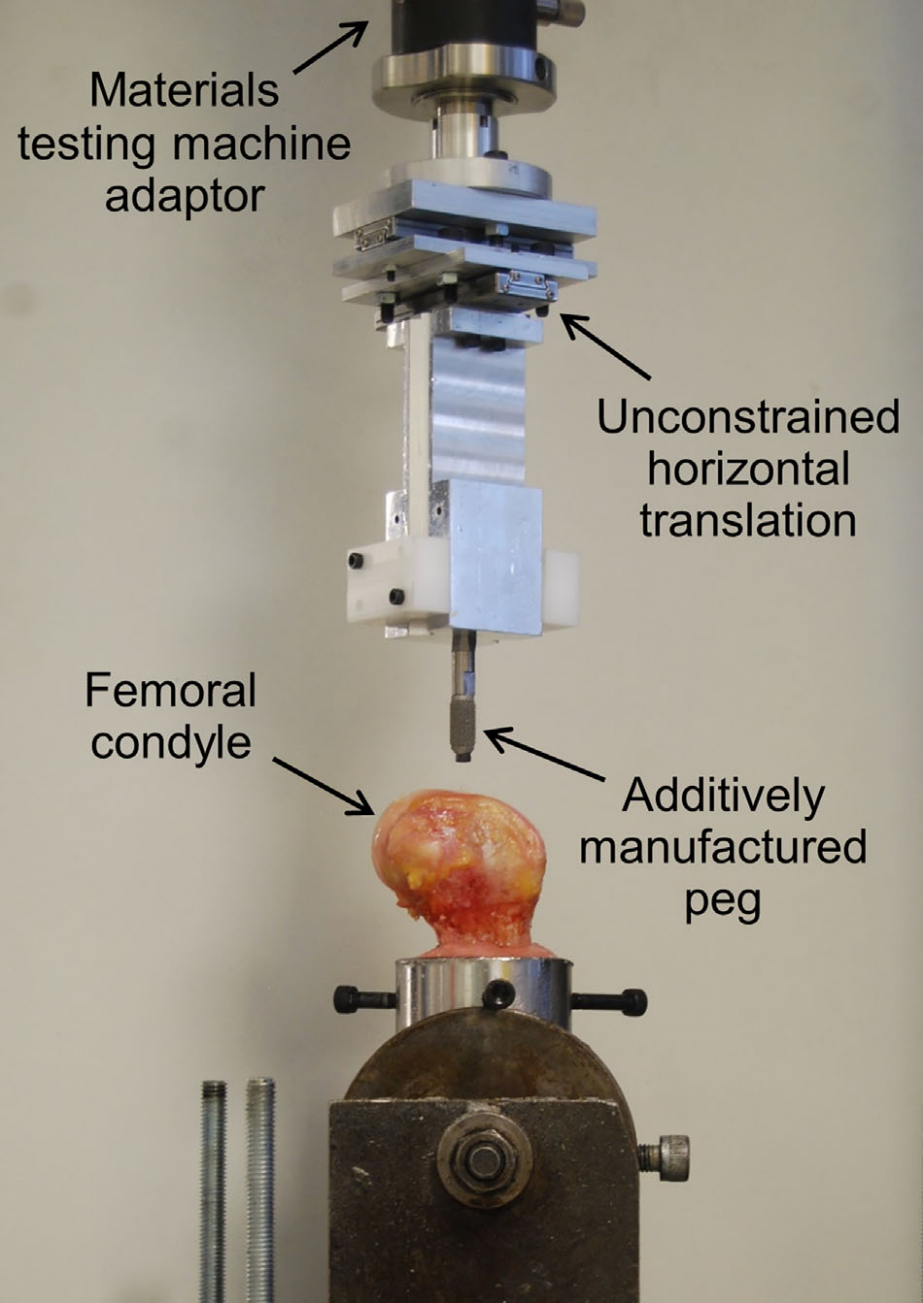
The testing set‐up for the cadaveric knee condyle tests.

The prepared femora were then mounted into a testing rig in the materials testing machine (Fig. 4). Pegs were then pushed in at 1 mm/s, before being pulled out at 1 mm/s after a 10 s pause.

### Statistical Analysis

Knee test data were analyzed in SPSS (v22, IBM, NY). Data were tested for normality using a Shapiro‐Wilk test and then analyzed with a two‐way analysis of variance (ANOVA) with the measured force as the dependent variable and the loading direction (in, out) and the peg type (optimized barbed fixation, optimized conventional) as the independent variables. The significance level was set to *p* *< 0.05*. Post hoc *t*‐tests with Bonferroni correction were applied when differences across tests were found. Adjusted *p*‐values, multiplied by the appropriate Bonferroni correction factor in SPSS, have been reported.

## RESULTS

### Design A: Solid Peg Optimization

**Figure 5:**
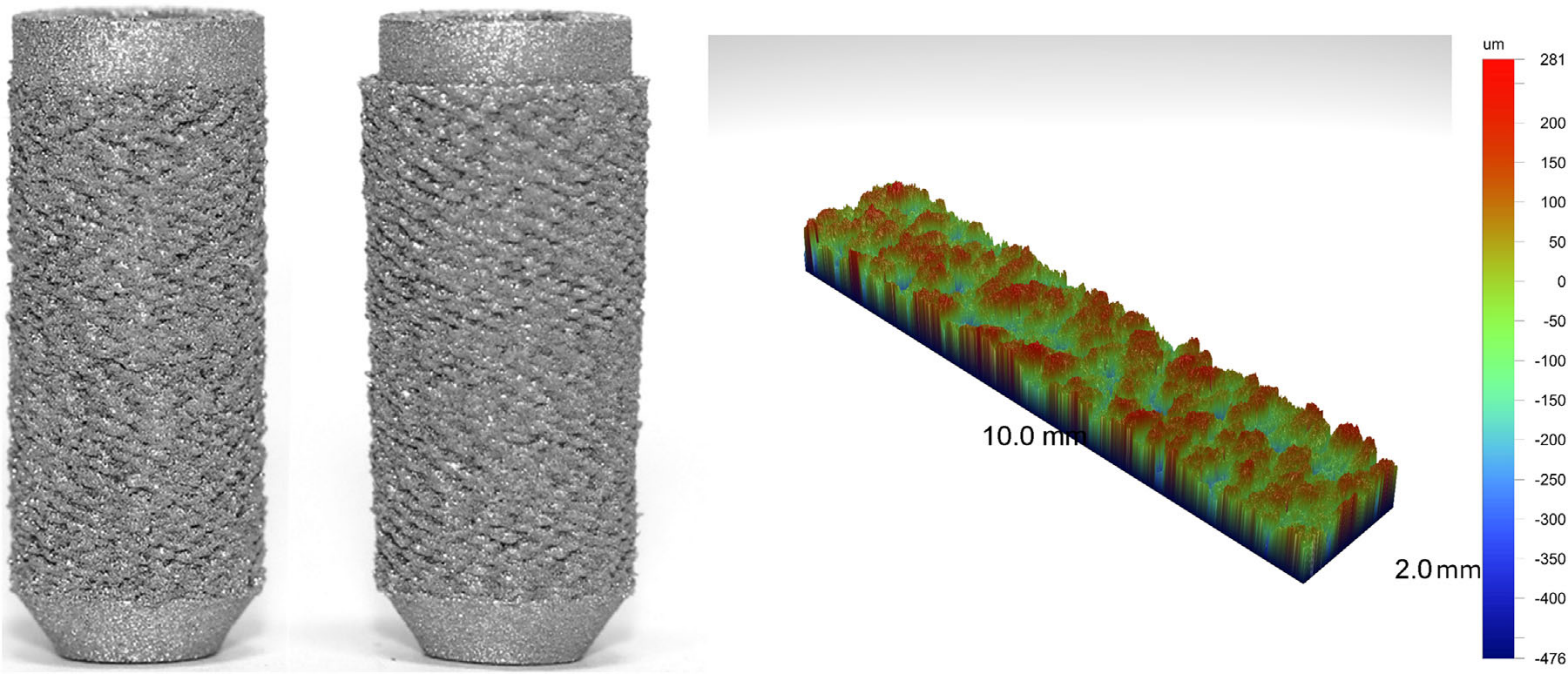
Straight sided (left) and tapered (middle) solid press‐fit pegs. An example surface roughness measurement (right) is also shown.

**Figure 6:**
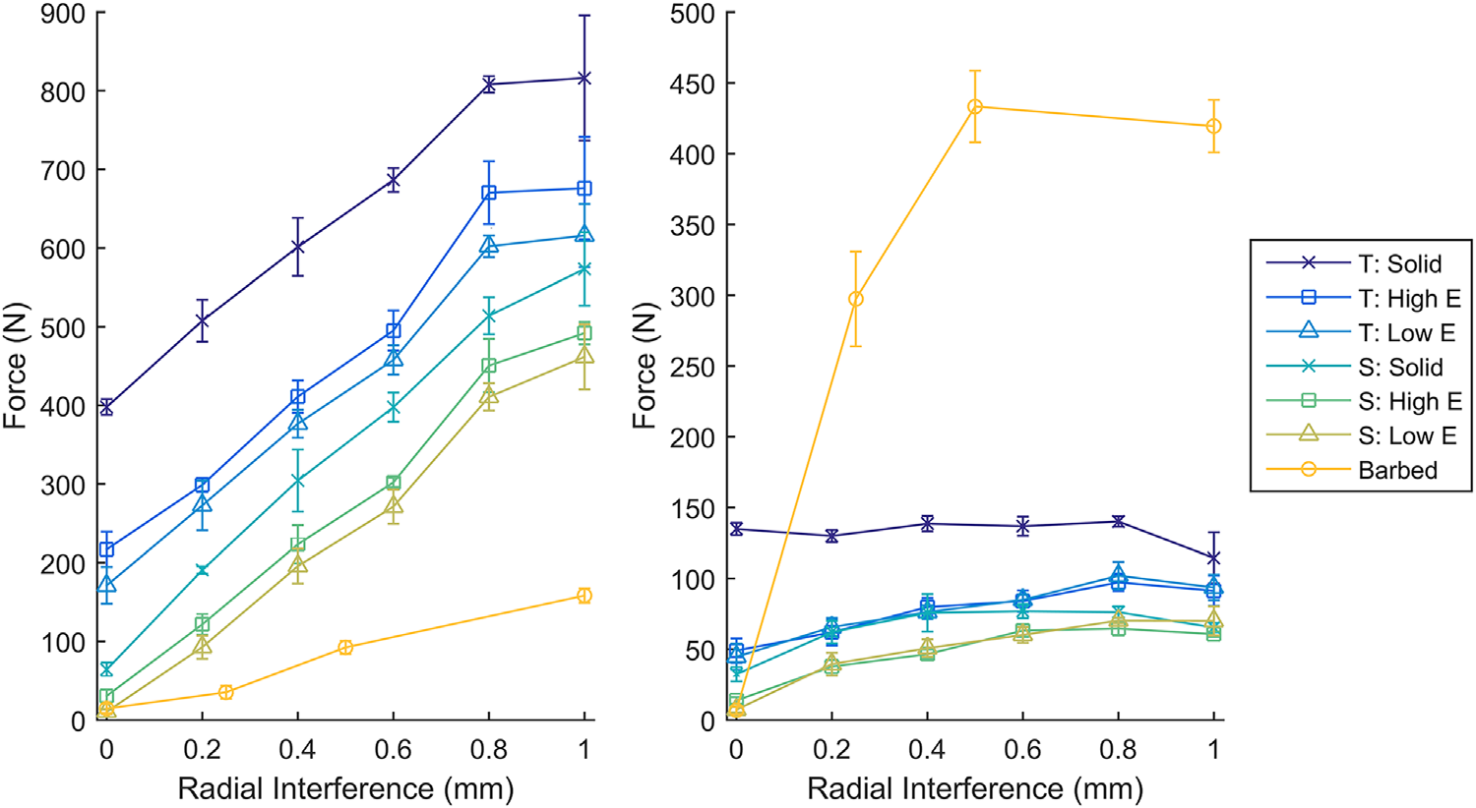
The force required for push in (left) and pull out (right) for different interference fits in synthetic bone (*N* = 4 per point). With increasing interference, push‐in force increased, while pull‐out force increased before reaching a maximum. T, Tapered cylinder; S, Straight sided cylinder; Solid, Solid implant; Low E, 600 MPa porous implant; High E, 2.6 GPa porous implant; Barbed, Baseline version of barbed fixation implant with varying outer length.

The desired roughness (Fig. 5) was successfully manufactured with *R*_z_ = 730 μm (the arithmetical mean deviation, *R*_a_, of the surface was 80 μm, and the root mean squared deviation, *R*_q_, was 100 μm). In the synthetic bone tests, as interference fit was increased, the push‐in force also increased for both the straight sided and tapered solid pegs (Fig. 6). However, pull‐out load initially increased with interference before reaching a maximum (Fig. 6, Table 2). The pull‐out/push‐in ratio decreased with increasing interference fit for all press‐fit peg design variants (Supplementary Fig. S2). The tapered peg required more insertion force, but also provided greater pull‐out resistance. Optimums are detailed in Table 2.

**Table 2:** Comparison of Different Press‐Fit Peg Types and the Optimum Level of Interference for Maximizing Pull‐Out Load

Peg	Tapered	Pull‐out Maximum ± S.D. (N)	Radial Interference at Maximum (mm)	Force Ratio (Out/In) at Maximum
Solid	Yes	140 ± 4	0.8	0.17
	No	77 ± 5	0.6	0.20
High Modulus Porous	Yes	97 ± 6	0.8	0.15
	No	65 ± 5	0.8	0.15
Low Modulus Porous	Yes	102 ± 10	0.8	0.17
	No	70 ± 2	0.8	0.17
Barbed Fixation (Baseline)	No	433 ± 25	0.5	4.69

### Design B: Porous Peg Optimization

**Figure 7:**
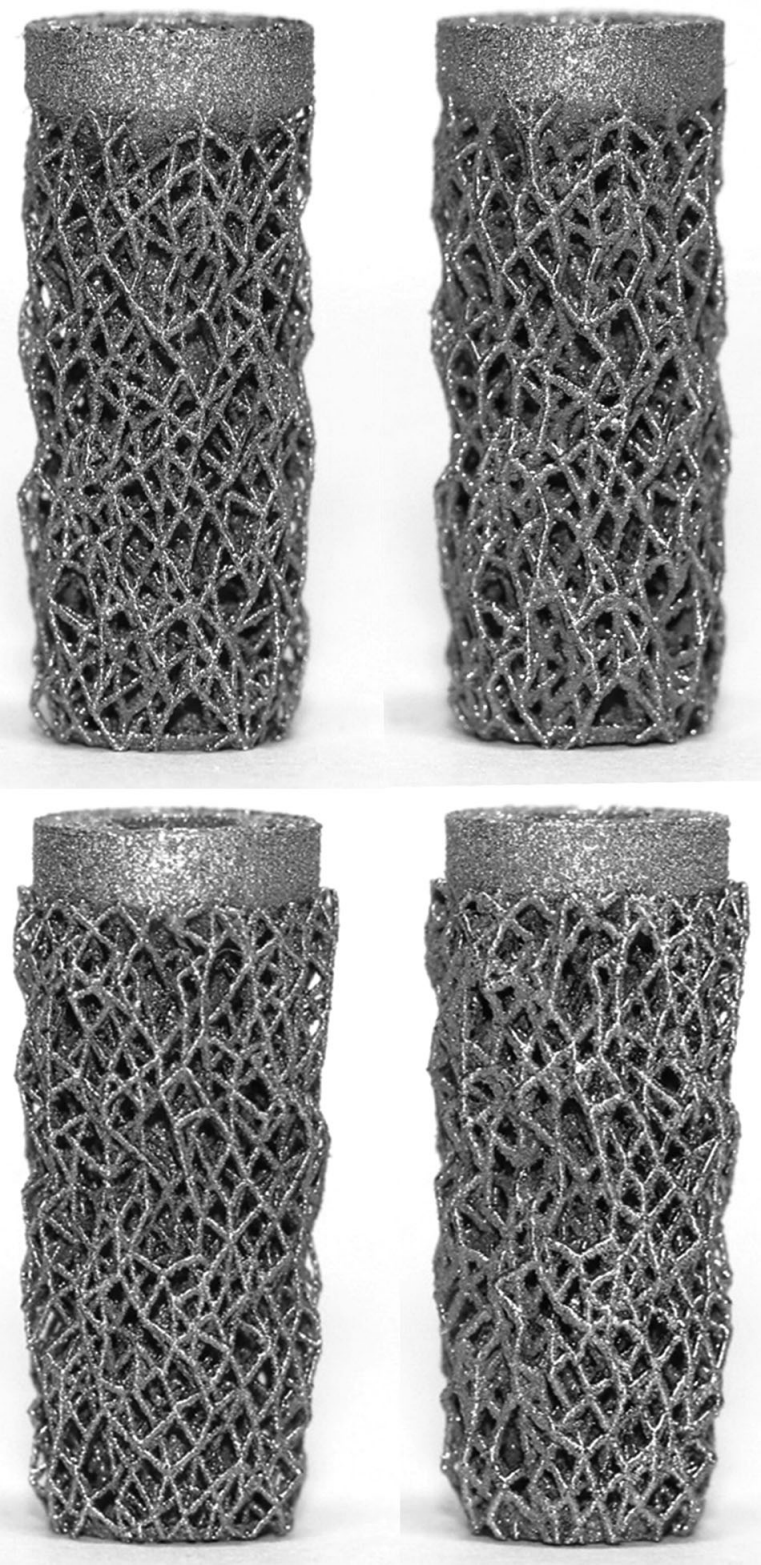
Low effective modulus (left) and high effective modulus (right) straight sided (top) and tapered (bottom) porous press‐fit pegs.

The target modulus values for the porous pegs were successfully manufactured (Table 3, Fig. 7). As with the solid pegs, increasing interference increased the push‐in force and also increased pull‐out force until it reached a maximum value (Fig. 6, Table 2). Again, the tapered structure provided more pull‐out resistance but required a greater insertion force and, as was the case for design A, the pull‐out/push‐in ratio decreased with increasing interference (Supplementary Fig. S2). The modulus of the structure had little influence on the pull‐out load (Fig. 6) but the lower modulus structure was easier to insert, requiring 30–70 N less force (Fig. 6). Optimum values of interference fit are detailed in Table 2.

**Table 3:** Mean ± S.D. Mechanical Properties of the Stochastic Porous Structures

Modulus	Shape	Porosity (%)	Modulus (GPa)	Strength (MPa)
High	Straight	79.4 ± 0.2	2.63 ± 0.06	36.7 ± 1.4
	Tapered	80.0 ± 0.1	2.56 ± 0.05	34.2 ± 0.7
Low	Straight	89.2 ± 0.1	0.63 ± 0.03	8.7 ± 0.1
	Tapered	89.5 ± 0.1	0.59 ± 0.02	7.9 ± 0.1

### Design C: Barbed Fixation Optimization

**Figure 8:**
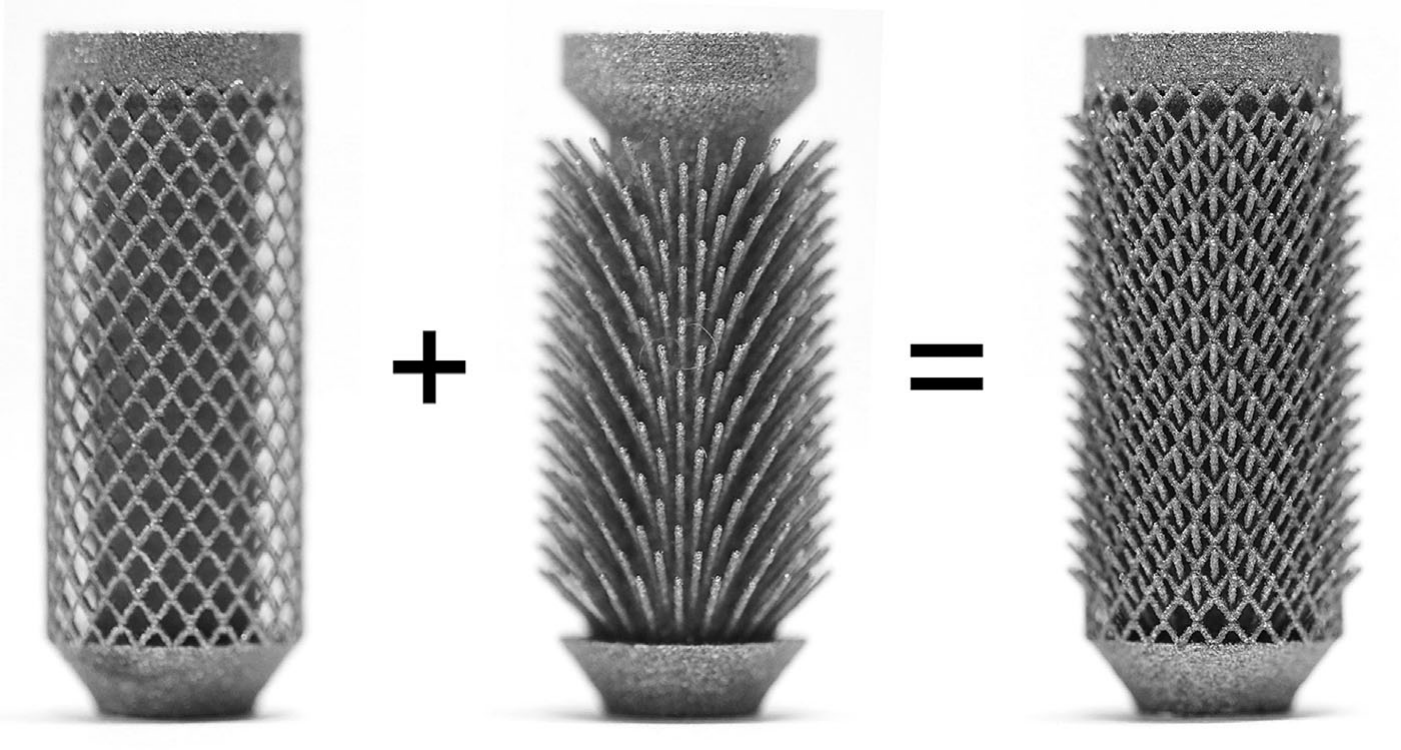
The optimized barbed fixation specimen deconstructed (left and middle) to demonstrate how the design works, and as tested (right).

Video analysis confirmed that the barbed fixation mechanism functioned as intended: The struts flexed upon insertion, limiting synthetic bone damage, enabling them to grip onto the bone upon pull out (see video in supplementary material and Fig. 8).

**Figure 9:**
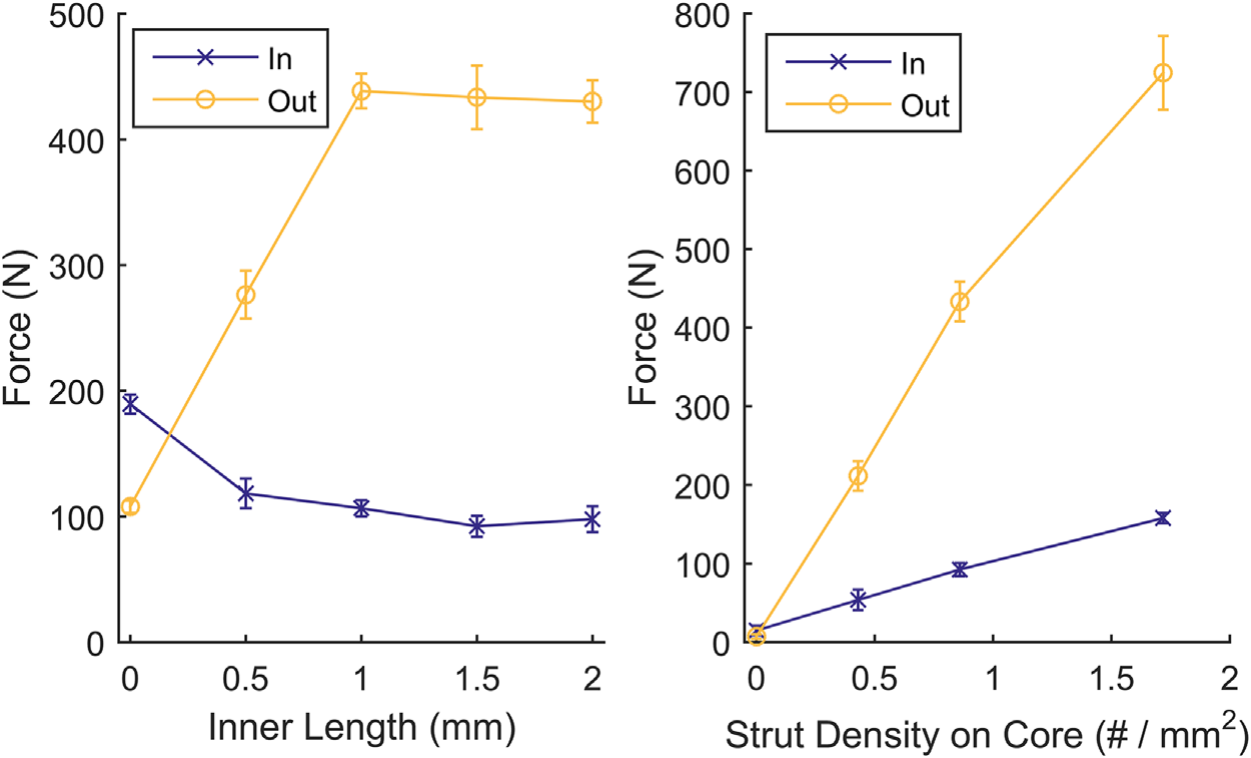
(a) Push‐in and pull‐out forces for different inner length barbed fixation designs. Allowing the struts to project from within the implant, through open pores, increased the pull‐out forces while decreasing the push‐in force; the maximum was reached through the strut hitting the cage. (b) Push‐in and pull‐out forces for increased strut density. Increasing the strut density effectively increased interference, increasing push in and pull out. *N* = 4 for all points, tests performed in synthetic bone.

All of the properties that influenced strut flexibility influenced the push‐in/pull‐out mechanics. The inner length in particular altered the ratio of forces: Without any inner length, the push‐in force was greater than the pull‐out load, as was the case for the solid and porous pegs. However, by projecting the strut from deep within the peg, the push‐in force was decreased while the pull‐out load increased (Fig. 9a) allowing for pull‐out/push‐in ratios >8 to be achieved (see Supplementary Fig. S2).

All other variables shown in Figure 1 were optimized as well: Increasing the outer length of the struts behaved in a similar manner to increasing interference fit for the solid and porous pegs: Push‐in force increased, with pull‐out force increasing before reaching a maximum (Fig. 6, Table 2). Increasing the number of struts also effectively increased the interference, with more struts requiring greater push in, but also providing greater pull‐out resistance (Fig. 9b). Thickness and angle were also found to have maximums at 0.3 mm and 45°, respectively.

Variables resulting in maximum pull‐out loads (inner length = 1.5 mm, outer length = 0.5 mm, angle = 45°, thickness = 0.3 mm, and strut density = 1.7 per mm^2^) were combined to manufacture the optimized barbed fixation peg (Fig. 8).

### Optimized A Versus B Versus C Versus Screw

**Figure 10:**
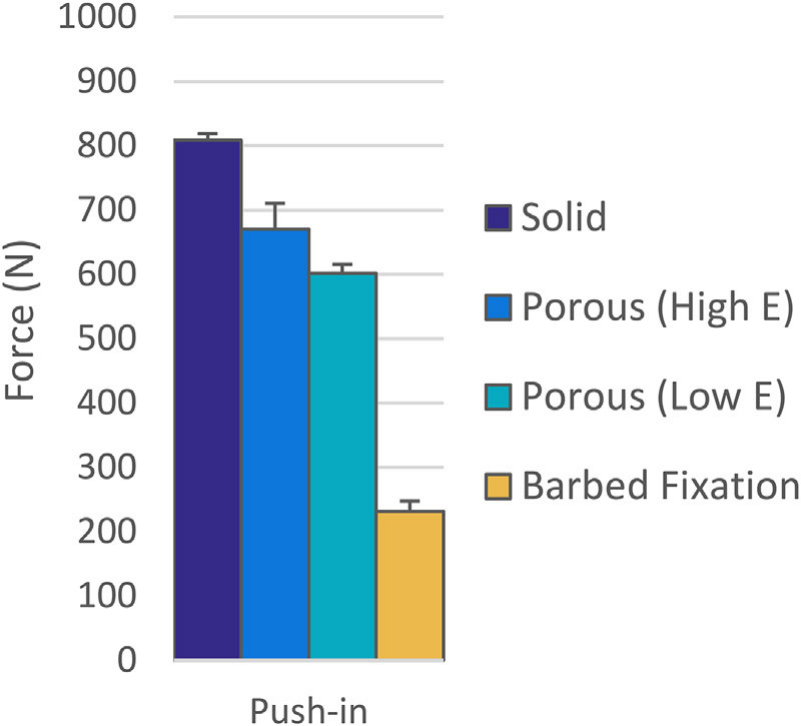
Push‐in force comparison for optimum the barbed fixation design and the tapered press‐fit pegs with their optimum interference in synthetic bone (*N* = 4 per design). The barbed fixation more than halved push‐in forces.

**Figure 11:**
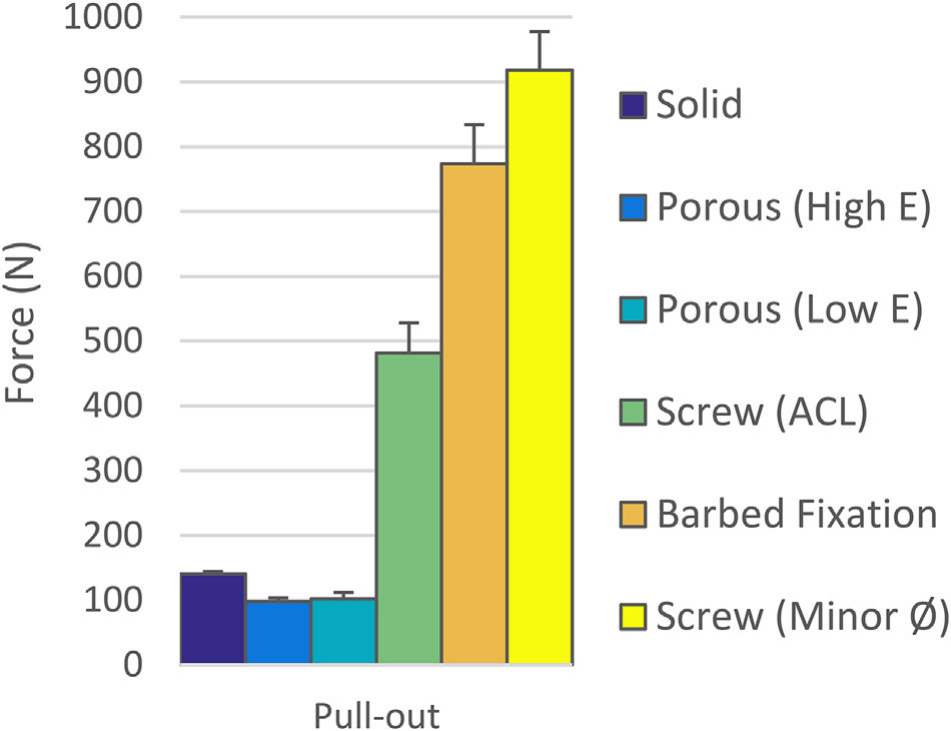
Pull‐out force comparison between the optimum barbed fixation design, the tapered press‐fit pegs with their optimum interference and an interference screw at the recommended hole size for ACL surgery and with a hole sized to its minor diameter in synthetic bone (*N* = 4 per design). The barbed fixation surface required >5× the pull out compared to press‐fit pegs and achieved pull out comparable to an interference screw.

From the data in Figures 6 and 9, the solid, porous and barbed fixation implants with the highest pull‐out force were selected. These were tapered side walls variants for Design A and B (Table 2), and for Design C the best combination of strut inner length, outer length, angle, thickness, and density (Fig. 8). Push‐in/pull‐out testing on these designs showed that the barbed fixation surface provided both the lowest push‐in force (Fig. 10) with the highest pull‐out force (Fig. 11). The barbed fixation surfaces provided 3× more pull‐out force than push‐in force whereas pegs relying on interference fit alone were only able to provide between 0.15 and 0.2× the push in (Table 2). This meant that the barbed fixation technology was effectively able to invert traditional push‐in/pull‐out fixation mechanics, such that its pull out was equivalent to a press‐fit pegs’ push in, and its push in equivalent to a press‐fit pegs’ pull out.

As would be expected, the interference screw vastly outperformed solid and porous peg designs that relied on press‐fit alone. However, the optimum barbed fixation surface achieved screw‐level fixation, exceeding that of the screw at its recommended interference for ACL surgery, and approaching the maximum possible for the screw (Fig. 11).

### Human Bone Tests

Having achieved the maximum measured pull‐out loads in the synthetic bone tests, the optimum barbed fixation design, with a Ø8 mm hole (Fig. 8), and the tapered solid peg, with a Ø6.4 mm hole, were selected for cadaveric testing.

**Figure 12:**
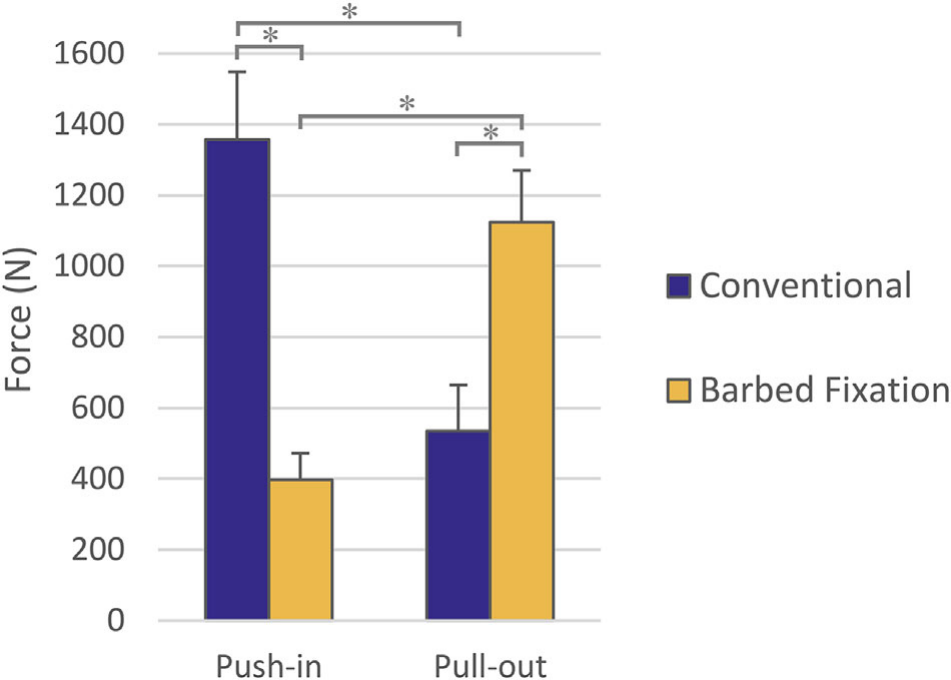
Comparison between optimized conventional and barbed fixation peg designs in cadaveric knee condyles (*N* = 8 per design). The human bone tests confirmed results from the sawbones model: The barbed fixation design offered higher pull‐out force for less push‐in effort. An asterisk (*) indicates *p* < 0.001.

The trends for the cadaveric tests replicated those seen in the synthetic bone tests. Differences between push‐in and pull‐out forces were affected by the peg design while the force generated by each design depended on loading direction (ANOVA interaction *p* < 0.001). For the barbed fixation peg, the pull‐out force was 2.8× the push‐in force (Fig. 12, mean difference 726 N, 95% confidence interval 581–871 N, *t*‐test *p* < 0.001) whereas for the optimized conventional technology, the pull out was only 0.4× the push in (Fig. 12, difference −822 N, 95% confidence interval −967 to −677 N, *t*‐test *p* < 0.001).

Comparing the peg designs, during push in, the optimized conventional peg required mean 959 N more force to insert than the barbed fixation design (Fig. 12, 95% confidence interval 814–1,104 N, *t*‐test *p *< 0.001), whereas during pull out, the barbed fixation peg provided mean 589 N more pull‐out resistance (Fig. 12, 95% confidence interval 444–734 N, *t*‐test *p* < 0.001). Indeed for all tests, in all femora, in all condyle positions, the minimum pull‐out force measured for the barbed fixation (918 N) exceeded the maximum measured for the rough peg (794 N).

## DISCUSSION

The most important finding of this study was that push‐fit orthopaedic implants can be designed and manufactured to anchor in bone with screw‐level fixation, greatly improving on conventional press‐fit technology. This barbed fixation technology complements recent advances in long‐term fixation offered by open porous structures for bony ingrowth^18^, ^19^ by providing a mechanism to also improve the initial fixation of implants. Indeed, the two technologies are ideally applied together as it was found that projecting a fixation feature from deep within the implant, through a pore, the length and thus flexibility of the strut increase offering a greater initial fixation advantage. Through doing this, it was possible to design for the first time a push‐fit orthopaedic fixation surface that was easier to insert than to remove, halving the insertion force while doubling the anchoring forces compared to conventional press‐fit technology. This offers a two‐fold advantage: First, lower insertion force requires less implant impaction while guaranteeing fixation, benefiting minimally invasive/robotic surgery and lowering impaction fracture risk. Secondly, the increased anchoring force offers new options for less invasive, smaller implant designs utilizing low profile fixation. Combined with the inherent advantage of push fit technology, not requiring rotational symmetry/modularity, barbed fixation could simplify existing procedures while also enabling new treatment paradigms.

The large differences in results from the press‐fit and barbed fixation technologies can be explained by their differing fixation mechanisms. Conventional press‐fit technology relied on friction, generated by either a high friction coefficient from a scratch‐fit, or a high reaction force from hoop stresses generated in the bone.^9^, ^15^, ^34^, ^35^ These mechanisms are limited by permanent bone deformation upon insertion^34^, ^35^ and by stress relaxation over time.^9^ The barbed fixation however relied less on friction, rather the struts flexed away from the bone upon insertion, as demonstrated by the lower push‐in force (Figs. 10 and 12) and supplementary material video, and anchored the fixation features under intact trabeculae. Removal of the implant therefore required localized cancellous bone fracture, mimicking screw fixation which also required fracture/shearing to remove the implant, greatly increasing the pull‐out load compared to friction forces alone. By not relying on traditional press‐fit mechanics, the fixation technology may also be less reliant on accurate bone surface preparation and so could be less sensitive to variations in surgical technique.

The level of pull out achieved by the barbed fixation in both synthetic and human bone (mean ± S.D. of 773 ± 61 N and 1124 ± 146 N, respectively) is of the same magnitude as that measured for a variety of bone screws in synthetic (Fig. 11)^5^, ^7^, ^17^, ^40^ and cadaveric bone.^6^, ^17^ It greatly exceeds current additive manufactured knee replacement designs which have peg fixation of only 100 N;^16^ applying the technology to such designs, or equivalent components in the shoulder, could offer clinical benefit through overcoming lift‐off^42^ or rocking^43^, ^44^ loosening mechanisms. Other researchers have also found that additive manufactured fixation features could influence initial implant stability.^14^, ^15^ They showed rigid surface features could increase implant stability through a scratch fit increasing friction compared to a porous or rough implant surface. Our study showed that by projecting the fixation feature from within the implant, further improvement to implant stability can be gained, both lowering the push‐in and increasing the pull‐out force compared to surface features alone (Fig. 9a), as the fixation methodology changed from a scratch‐fit, to an anchoring fixation. A similar trend, that low stiffness flexible features improve initial fixation, has also been recently observed for ultra‐high molecular weight polyethylene pegs.^45^ In our study, once the titanium alloy struts were flexible enough to bend during push in/pull out, further gains in stability offered by increasing inner length were then limited by the porous cage. During pull out, this contact with the porous cage was beneficial as it shortened the struts effective length, increasing their stiffness and thus their pull‐out resistance. However, further increases to inner length did not affect this strut shortening mechanism resulting in the observed plateau in pull‐out force (Fig. 9a).

Our study found that all conventional press‐fit peg variants had a maximum possible pull‐out load: Permanent bone deformation limited the effective interference despite higher nominal interference.^34^, ^35^ Interestingly, reducing the effective modulus of the implant to levels equivalent to trabecular bone did not alter this finding as demonstrated by the low modulus porous peg results offering only marginal gains compared to the high modulus porous structure. The tapered shape however did result in larger pull‐out forces. This was likely because for cylindrical pegs, the deeply inserting portions of the peg damage the bone surface for the more proximal portions, whereas for the taper, each part of the peg can engage with undamaged bone. A previous cadaveric study in the femoral condyles, evaluating commercially available press‐fit pegs, found a maximum pull‐out force of 150 N with 0.8 mm radial interference.^9^ Our synthetic bone results were near identical (Table 2) and we found higher pull‐out forces when testing in the femoral condyles. This indicates that our additive manufactured rough surface achieved equivalent/superior initial fixation to clinical technology.

In vivo, a critical goal for initial implant stability is to limit the amount of small micromotion experienced under cyclic loading. It has been suggested that 40–150 um is tolerable, while values in excess of this lead to fibrous tissue formation rather than bony ingrowth.^46^, ^47^, ^48^, ^49^, ^50^, ^51^ In this study, micromotion was not assessed as it would have required more lengthy cyclic loading and narrowing the large design space (>600 samples) with such a test was deemed impractical. Future work might seek to test the optimum designs, and those close to the optimum, in micromotion tests. It was also assumed that the individual design variants maximizing pull‐out force could be combined to make an optimized barbed fixation design. While indeed the optimized design achieved the highest pull‐out load of any sample, this does not rule out the possibility that there could have been some negative interaction when combining individually established optimums. The device also likely works best when tuned to the bone properties, for example, struts that are too stiff/strong compared to the surrounding bone will not flex away from it upon insertion, but will deform/damage the bone like a conventional press‐fit. Conversely, struts that are too flexible/weak will fail at a load lower than the bone and therefore implant failure would limit fixation strength. In this study, the performance tuning of the designs was conducted in a synthetic bone which may mean that they were optimized for a bone that may be stronger, or weaker than that for specific applications. We used the highest strength cellular foam available from a common biomechanical testing materials supplier^52^ and found that the human bone tests exhibited both higher push‐in and pull‐out forces suggesting that the synthetic bone was weaker than that in the femoral condyles. The properties of bone vary greatly around the body^53^ and are dependent on age.^54^ Given the ease with which parameters such strut thickness can be altered during the additive manufacturing process without having to change the CAD designs, future work may slightly tweak the optimums established in this paper when applying the technology to a specific implant or patient demographic. Similarly, the technology could be re‐optimized for different metal alloys such as cobalt‐chrome, or even for polymers. Our study did not investigate if fretting could occur between the struts and the cage, nor did it evaluate methodology to clean implants or mitigate strut breakages. Cleaning methods have already been developed by industry as additive manufactured implants utilizing porous structures have already been implanted in patients worldwide.^12^, ^13^, ^21^ Strut breakage occurred during testing, though was very design dependent: Some variants lost nearly all their struts during pull out, while other designs remained intact. Generally, non‐broken struts were observed to have bent around the porous cage and therefore switching to a more ductile metal, such as commercially pure titanium, would likely reduce the risk of strut breakage. With the high levels of fixation offered by the barbed fixation design, revision could also be a concern for surgeons as the fixation is so good, the implant could be too hard to remove in cases such as infection. This is also a challenge for open porous implants that have achieved bony ingrowth or for damaged screws, and therefore similar revision techniques, such as using a trephine, could be adopted for the barbed fixation technology. An advantage is that the barbed fixation provided higher levels of stability than a press‐fit peg meaning that lower profile, and more bone preserving, fixation could be designed thereby improving revision options for open porous additive manufactured implants that have achieved bony ingrowth.

In conclusion, this paper examined three ways in which additive manufacturing technology could influence the initial fixation of implants: Through press‐fit roughened solid, press‐fit porous, or barbed fixation surfaces. It found that by designing directionally biased fixation features, projecting them through open pores to increase their flexibility, the mechanics of initial fixation could be dramatically improved allowing screw‐level fixation from push fit implant design. This step change in implant fixation technology provides exciting new options for surgical interventions that require metallic implants to fixate in bone.

## AUTHORS' CONTRIBUTIONS

Research design, manufacturing, data acquisition, analysis and interpretation, drafting the manuscript by RvA. Research design, manufacturing, critically revised manuscript by SG. Data acquisition and analysis, critically revised manuscript by PM. Research design, data interpretation, critically revised manuscript by JJ. All authors have read and approved the final submitted manuscript.

## Supporting information

Additional supporting information may be found in the online version of this article at the publisher's web‐site.

Supporting Video S1.

Supporting Figure S1.

Supporting Figure S2.
