# Supplementary figures and images for: Additive manufactured push‐fit implant fixation with screw‐strength pull out
Source: J Orthop Res. 2017 Nov 22;36(5):1508–18. doi: 10.1002/jor.23771 (PMC6175131; doi:10.1002/jor.23771)

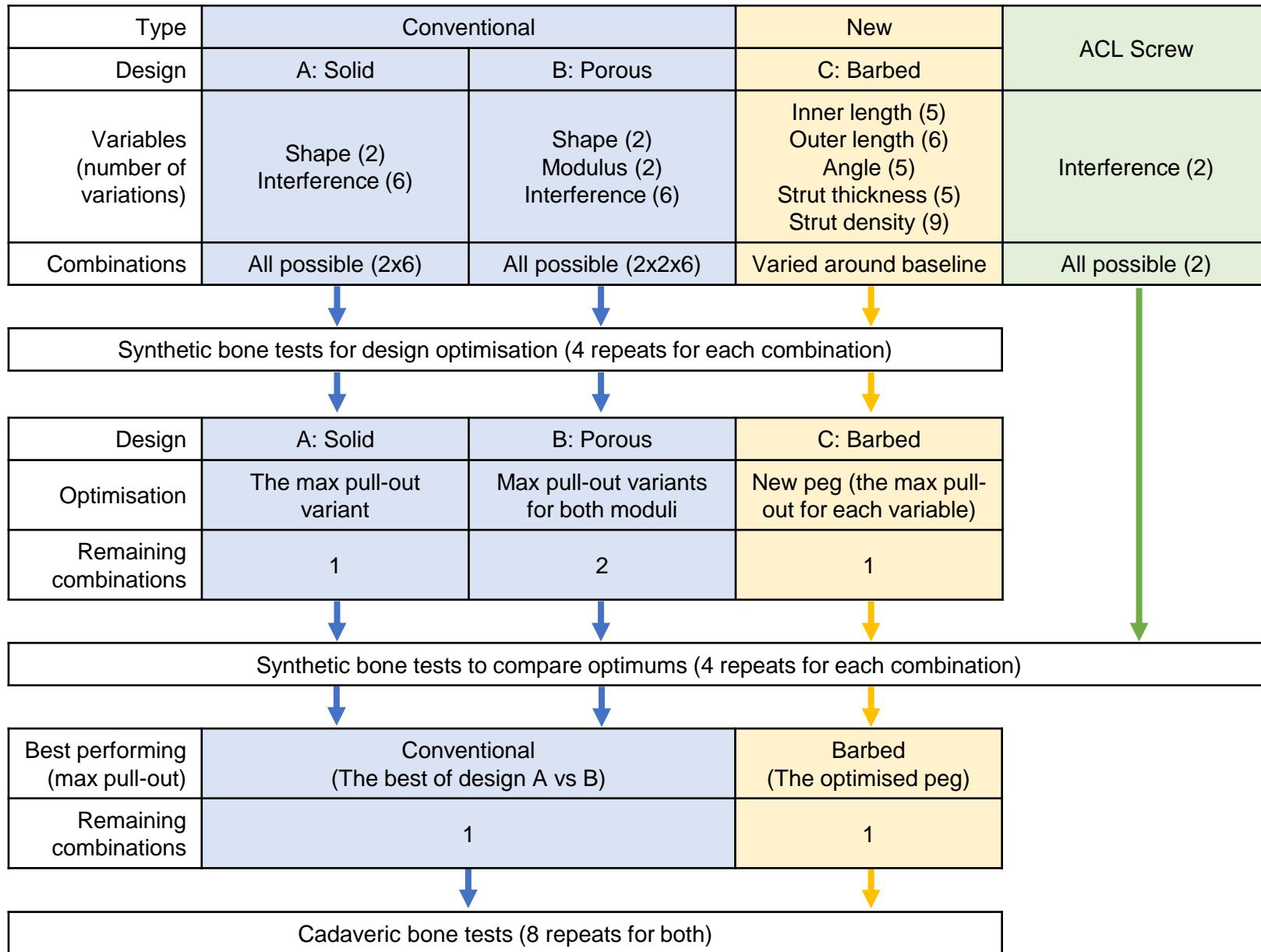

Figure S1: An overview of the tests performed

Supplement: Supplementary file 2 — Supporting Figure S1. [file JOR-36-1508-s002.pdf]
