# Supplementary material for: Additive manufactured push‐fit implant fixation with screw‐strength pull out
Source: J Orthop Res. 2017 Nov 22;36(5):1508–18. doi: 10.1002/jor.23771 (PMC6175131; doi:10.1002/jor.23771)
Supplement: Supplementary file 3 — Supporting Figure S2. [file JOR-36-1508-s003.pdf]

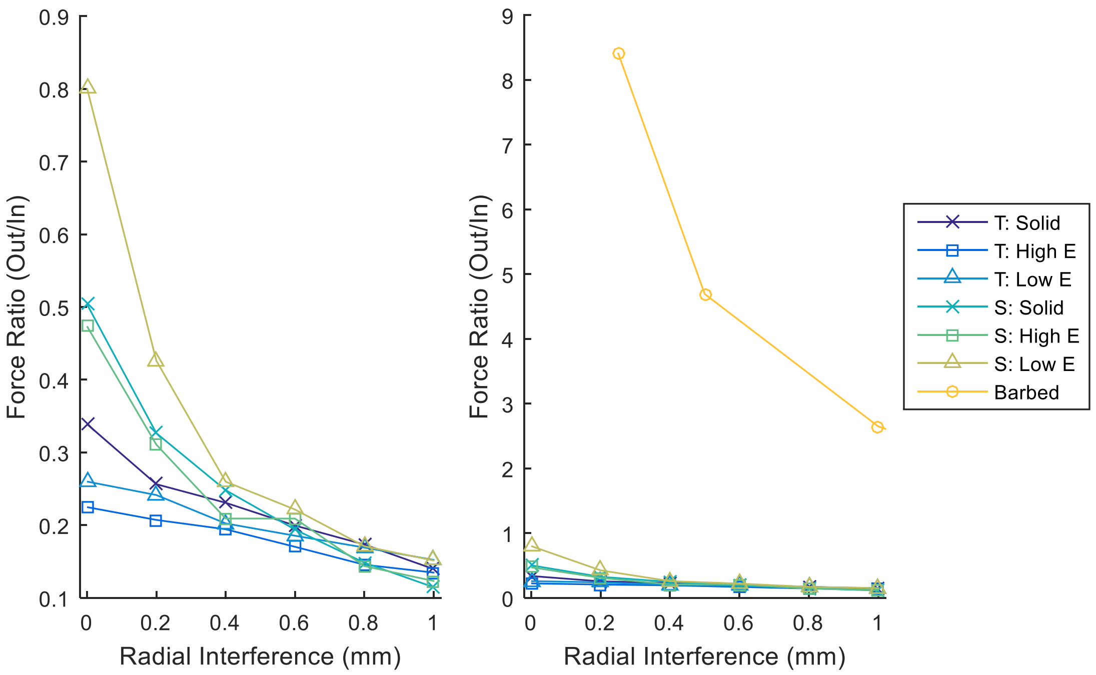

Figure S2: The change in pull-out/push-in force ratio for different interference fits in synthetic bone (N=4 per point). Left) Comparison of conventional technologies only, it can be seen that the force ratio decreased with increasing interference indicating that increasing interference resulted in bigger increases in push-in force than pull-out force. Moreover, all ratios were less than 1 indicating push-in > pull-out for all combinations. Right) the same comparison of conventional technologies, but with the barbed fixation also shown (note the factor 10 increase in the y-axis scale). The barbed fixation achieved force ratios much greater than 1, indicating pull-out > push-in. The trend for decreasing ratio with increasing interference was also observed for the barbed design. T: Tapered cylinder, S: straight sided cylinder, Solid: solid implant, Low E: 600 MPa porous implant, High E: 2.6 GPa implant, Barbed: baseline version of barbed fixation implant with varying outer length.
